# Radiodiagnostic Imaging in Pregnancy and the Risk of Childhood Malignancy: Raising the Bar

**DOI:** 10.1371/journal.pmed.1000338

**Published:** 2010-09-07

**Authors:** Eduardo L. Franco, Guy-Anne Turgeon

**Affiliations:** 1 Division of Cancer Epidemiology, McGill University, Montreal, Canada; 2 Division of Radiation Oncology, McGill University, Montreal, Canada

## Abstract

Eduardo Franco and Guy-Anne Turgeon discuss new findings from Joel Ray and colleagues on the cancer risk following prenatal exposure to radiodiagnostic imaging, and where new research needs to be focused.

**Linked Research Article**

This Perspective discusses the following new study published in *PLoS Medicine*:

Ray J, Schull M, Urquia M, You J, Guttman A, et al. (2010) Major radiodiagnostic imaging in pregnancy and the risk of childhood malignancy: A population-based cohort study in Ontario. PLoS Med 7: 337. doi:10.1371/journal.pmed.1000337

In a record linkage study, Joel Ray and colleagues examine the association between diagnostic imaging during pregnancy and later childhood cancers.

## Setting Limits to Risk: *Primum non nocere* (Now and Later)

Determining carcinogenic risk following imaging radiation exposure is based mostly on predictive mathematical models developed from empirical data provided by large observational studies, such as the large cohort study of Japanese atomic bomb survivors [1]. The United Nations Scientific Committee on the Effects of Atomic Radiation (UNSCEAR) model developed by the United Nations [2] and the Biological Effects of Ionizing Radiation (BEIR) VII model developed by the US National Research Council [3] have been validated for risk assessment using smaller exposed cohorts, for example, those of nuclear plant workers, the Chernobyl population, workers exposed occupationally, and patients undergoing medical procedures involving radiation exposure. In general, these models have been used to establish maximum acceptable levels of exposure and protection guidelines in occupational environments and for setting limits and safety standards in radiodiagnostic and therapeutic exposures [4]. In this issue of *PLoS Medicine*, Joel Ray and colleagues report a large study from Ontario, Canada that adds to our growing understanding of cancer risk following prenatal exposure to radiodiagnostic imaging [5].

## Issues with Observational Studies

The possibility that pre- or postnatal exposure to radiation from diagnostic imaging procedures increases cancer risk is among the most controversial topics in medicine [6]–[8]. The association of in utero irradiation and increased risk of childhood malignancies has been studied since the 1950s. Ionizing radiation has been shown to cause leukaemia and solid tumours in both exposed adults and children [1],[7],[9]. However, for in utero exposure the magnitude of the risk from low-dose radiation and whether risk varies throughout pregnancy have been open to debate. Some supporting empirical data exist for the increased risk of leukaemia [10], but the issue is far from resolved despite numerous case-control and cohort studies, and in spite of meta-analyses that have attempted to provide average risk effects [4],[11].

The lack of a clear-cut picture rests on a number of practical issues. First, the average radiation dose from individual diagnostic or therapeutic procedures has historically declined owing to improvements in the technology and equipment safeguards. Although the number of imaging procedures has increased over the years, equipment efficiency has gradually improved. As a consequence, more recent studies that collected data based on plain films with lower exposure doses are less likely to detect associations than first generation investigations. For computerized tomographic (CT) scans, doses are typically higher than those for ordinary plain films and, although efficiency has improved, the multiple slices commonly taken in helical scans subject patients to relatively higher overall doses than those from first-generation plain films. Second, childhood malignancies are very rare; thus individual studies tend to lack the necessary precision to detect low-level associations. A case in point is the observation that among some 800 atomic bomb survivors with prenatal exposure, there were only two cancer cases despite the prediction that 5–14 extra deaths due to childhood cancers were to be expected [12]. A third limitation in many observational studies is the lack of accurate exposure information with consequent misclassification of the exposure dose and attenuation of the associations. Having anatomically relevant dosimetry data is essential for interpreting findings. Calculation of relative risks (RR) separately for radiation exposure to the abdomen and for other body parts provides insights as to the validity of the associations as genuine causal relations [13]. In some case-control studies, reliance on subject recall for exposure ascertainment may lead to biases towards positive associations. Perhaps not surprisingly, for in utero exposure, studies showing a statistically significant positive association between diagnostic radiation and increased childhood cancer risk have been case-control studies. No cohort study has been able to show the same relationship [4],[6],[11].

One would expect that meta-analyses on the topic of prenatal exposures to radiation for diagnostic imaging purposes would have provided comparable findings. The International Commission on Radiation Protection (ICRP) concluded from the compilation of seven case-control and four cohort studies that there is a statistically significant increase in risk for all cancers (RR = 1.40, 95% confidence interval [CI] 1.23–1.59) and for leukaemia, specifically (RR = 1.30, 95% CI 1.16–1.46), but a weaker risk effect for solid tumours (RR = 1.14, 95% CI 0.94–1.40) [4]. A Cochrane systematic review conducted in 2008, which included only studies published between January 1990 and December 2006 (19 case-control and six cohort studies), did not find an association between prenatal exposures and leukaemia (RR = 0.99; 95% CI 0.78–1.13) and found at most weak effects for solid tumours [11]. Interestingly, another meta-analysis covering the same time span of published studies found a weighted mean RR of 1.16 (95% CI 1.00–1.36) for leukaemias, possibly as a result of more judicious weighting of abdominal exposures, whenever available in the published studies [14].

## Record Linkage to the Rescue

It is against the above backdrop of findings spanning multiple eras of exposure dose that the innovative study by Joel Ray and colleagues [5] in this issue of *PLoS Medicine* tries to advance our understanding of this important public health concern. The authors used a meticulous record linkage of administrative and health care utilization databases to identify all 1.8 million mother–child pairs from 1991 to 2008 in Ontario, Canada. They identified all term obstetrical deliveries and newborn records, and inpatient and outpatient major radiodiagnostic services, and linked these data to the Ontario tumour registry records to identify children who developed a malignant tumour after birth. There were four cancer cases among the children of 5,590 mothers having received any form of radiodiagnostic imaging procedure during pregnancy, versus 2,539 cases documented among the offspring of 1,829,927 mothers with no exposure. This translates into a RR (with pregnancies as denominators for rates) of 0.52. Most importantly, however, the authors analyzed the risk effect on the basis of the accrued person–time denominators for all children. This resulted in a crude hazard ratio [HR] of 0.69 (95% CI 0.26–1.82). Adjustment for a priori confounders, such as maternal age, income, urban status, maternal cancer, infant sex, history of chromosomal or congenital anomalies, and radiodiagnostic exposure after birth, did not materially change the association (HR = 0.68, 95% CI 0.25–1.80). Because their study was population-based and enrolled all eligible pregnancy–child pairs in the province during the accrual period, the incidence of childhood cancers observed among those with radiodiagnostic exposures represents a reduced burden of disease. For external validity purposes, however, the authors prudently concluded that, although the point estimate of the association was consistent with reduced risk, the statistical boundaries gauging the precision of the measure prevented them from ruling out a harmful effect from prenatal irradiation from medical sources. As an additional finding, the authors showed that radiodiagnostic testing increased about 6-fold, from 1.1 to 6.3 per 1,000 pregnancies during the nearly two decades covered by the study, with nearly three-fourths of these procedures being CT scans.

The Ontario study has several strengths related to the sheer size of the investigation, the fact that it was population-based, covered a relatively “modern” era of efficient and low radiation dose delivery to the target organs, had high-quality exposure ascertainment not collected via patient recall, and had sufficient follow-up for late childhood onset malignancies. Yet, the rarity of the outcome among exposed children prevented the authors from making more insightful analyses that could have revealed dose–response trends or permitted probing specifically for leukaemia risk. Likewise, with the low numbers of childhood malignancies, the authors could not examine the coherence of exposure-risk effects by anatomical site, thus comparing risk effects for exposures when the foetus is in the field of view (pelvis or abdominal) and when it receives at most scattered radiation, as is the case for imaging procedures involving only the extremities. As with most cohort studies, the Ontario investigation did not produce statistical evidence for an increased risk of childhood malignancies, but it provided important baseline data. It is also noteworthy that the Ontario investigation raised the bar substantially for future large-scale studies by showing the ways in which clever record linkage of multiple administrative and health care utilization databases can be used for cost-effective disease risk surveillance in a given setting.

## Stratifying Exposure-Risk Relations

The ongoing controversy about cancer risk following prenatal exposure to radiodiagnostic imaging has not been solved, but there seems to be a general consensus that diagnostic imaging poses a high risk of inducing childhood malignancies and that the null results must be interpreted carefully. Diagnostic CT imaging radiation involving the pelvis and the abdomen yields a high dose to the foetus [13],[15] and may thus, at least theoretically, increase the risk of childhood and even adult malignancies [9] relative to imaging procedures taken with the foetus outside of the field of view, which provides negligible scattered radiation exposure. Future studies should focus on accurately stratifying risk on the basis of this premise. We also believe that an international consortium that attempts to pool the data from the available investigations with the primary aim of categorizing exposure-risk associations on the basis of the magnitude of the dose delivered to the foetus could shed much light into this issue and assist policymakers in the future.

## Abbreviations

BEIR: Biological Effects of Ionizing Radiation
CI: confidence interval
CT: computed tomography
HR: hazard ratio
ICRP: International Commission on Radiation Protection
RR: relative risk(s)
UNSCEAR: United Nations Scientific Committee on the Effects of Atomic Radiation
